# Genome Sequence and Methylation Pattern of Haloterrigena salifodinae BOL5-1, an Extremely Halophilic Archaeon from a Bolivian Salt Mine

**DOI:** 10.1128/MRA.00275-21

**Published:** 2021-05-06

**Authors:** Priya DasSarma, Brian P. Anton, Satyajit L. DasSarma, Hedvig A. L. von Ehrenheim, Fabiana L. Martinez, Daniel Guzmán, Richard J. Roberts, Shiladitya DasSarma

**Affiliations:** a Department of Microbiology and Immunology, Institute of Marine and Environmental Technology, University of Maryland, Baltimore, Maryland, USA; b New England Biolabs, Ipswich, Massachusetts, USA; c Instituto de Investigaciones para la Industria Química, Consejo Nacional de Investigaciones Científicas y Técnicas, Universidad Nacional de Salta, Salta, Argentina; d Centro de Biotecnología, Faculty of Sciences and Technology, Universidad Mayor de San Simón, Cochabamba, Bolivia; e Blue Marble Space Institute of Science, Seattle, Washington, USA; Portland State University

## Abstract

The halophilic archaeon Haloterrigena salifodinae BOL5-1 was isolated from a Bolivian salt mine and sequenced using single-molecule real-time sequencing. The GC-rich genome was 5.1 Mbp, with a 4.2-Mbp chromosome and 5 plasmids ranging from 96 to 281 kbp. The genome annotation was incorporated into HaloWeb (https://halo.umbc.edu), and the methylation patterns were incorporated into REBASE (http://tools.neb.com/genomes/view.php?seq_id=99167&list=1).

## Abstract (precis)

The halophilic archaeon Haloterrigena salifodinae BOL5-1 was isolated from a Bolivian salt mine and sequenced using single-molecule real-time sequencing. The GC-rich genome was 5.1 Mbp, with a 4.2-Mbp chromosome and 5 plasmids ranging from 96 to 281 kbp. The genome annotation was incorporated into HaloWeb (https://halo.umbc.edu) and the methylation patterns into REBASE (http://rebase.neb.com).

Halophilic microbes capable of surviving extreme conditions are of interest for biotechnology and astrobiology (1–12). Our recent focus has been on high elevation and subsurface hypersaline environments, which yield polyextremophilic varieties. In this announcement, isolation of an extremely halophilic archaeon, Haloterrigena salifodinae BOL5-1, is reported, together with the first complete genome sequence for this species.

## ANNOUNCEMENT

Halophilic microbes capable of surviving extreme conditions are of interest for biotechnology and astrobiology (1–12). Our recent focus has been on high elevation and subsurface hypersaline environments which yield polyextremophilic varieties. In this announcement, isolation of an extremely halophilic archaeon, Haloterrigena salifodinae BOL5-1, is reported, together with the first complete genome sequence for this species.

Pink salt was sampled from a remote salt mine in the Department of Tarija, O’Connor Province, Bolivia (21°24′19.73″S, 64°07′51.52″W), at 1,230 m elevation, where temperatures range from −10 to 37°C. The salt samples were dissolved in CM^+^ medium and grown with shaking at 220 rpm at 37°C as previously described (13, 14). Enrichment cultures were plated onto CM^+^ agar plates, and a pigmented isolate, *H. salifodinae* BOL5-1, was purified by three rounds of streaking.

Nucleic acids were extracted using standard methods (14), and sequencing was performed using the Sequel platform (PacBio, Menlo Park, CA). A SMRTbell library was prepared from 5 μg unsheared BOL5-1 genomic DNA, size selected on the BluePippin system (Sage Science, Beverly, MA) with a lower limit of 15 kb, purified for three rounds with AMPure beads (Pacific Biosciences) at 0.45×, and sequenced on one single-molecule real-time (SMRT) cell with the Sequel binding kit version 3.0 with 20-h collection and 2-h preextension times. The sequencing subreads were filtered and assembled *de novo* using the microbial assembly pipeline under SMRTLink version 9.0.0.92188 with default parameters. The 121,750 mapped subreads (mean length, 14,289 bp; coverage, 340×) resolved into six polished, circular contigs.

The assembled *H. salifodinae* BOL5-1 genome sequence comprised 5,087,240 bp (GC content, 63.4%) and included a circular chromosome (4,180,318 bp; GC content, 64.7%) and the plasmids pHTS280.6 (280,619 bp; GC content, 61.9%), pHTS220 (220,397 bp; GC content, 62.2%), pHTS171 (171,484 bp; GC content, 64.2%), pHTS138 (138,030 bp; GC content, 55.7%), and pHTS96 (96,392 bp; GC content, 58.4%). The genes were predicted first using GeneMark HMM (15), analyzed further with HaloWeb version r1613245396 (16) and EMBOSS version 6.6.0.0 (17), and finally deposited in NCBI, where the genome was reannotated using NCBI’s Prokaryotic Genome Annotation Pipeline (PGAP) build 3190 (18). DTU Health Tech Feature Extract version 1.2 (19) was used to converge the GeneMark and GenBank annotations, and the genome sequence and annotation were made publicly available on HaloWeb (https://halo.umbc.edu).

The BOL5-1 genome contained 4,729 protein genes, plus 4 rRNA operons and 54 tRNA genes. The 16S RNA sequence and average nucleotide identity were used for taxonomic analysis at GenBank. The proteome was highly acidic (2), with a calculated mean pI value of 4.64, and nearly all of the core haloarchaeal orthologous groups (cHOGs) were present (20–22). The BOL5-1 genome contained expanded gene families, e.g., Orc/Cdc6, TATA-binding, and TFB protein genes (23), and a gene cluster for gas vesicle nanoparticles (24, 25) on the chromosome. The genome also encoded transposase genes, suggesting the presence of ISH elements (26).

Methylated DNA motifs and the methyltransferases (MTases) were identified using the Pacific Biosciences base modification analysis protocol under SMRTLink version 9.0.0.92188 using default parameters and were deposited in REBASE (Table 1) (27).

**TABLE 1:** Motifs containing the methylated bases ^m6^A and ^m4^C

Motif^*a*^	Modification type	No. of sites in genome	% detected	Mean IPD ratio^*b*^	No. of predicted ORFs^*c*^
GGRC**A**G	^m6^A	4,340	99.8	5.6	Unknown
CGTG**A**YC	^m6^A	3,713	100	5.1	Unknown
C**A**TTC	^m6^A	3,903	99.7	5.0	16,755 (M.Hsa51II)
GAGA**A**G	^m6^A	3,192	99.7	4.9	Unknown
ACGA**C**GC	^m4^C	4,012	88.5	3.3	Unknown
**C**TAG	^m4^C	4,856	74.5	3.2	18,220 (M.Hsa51I)

a Locations of methylated bases are in bold for the top strand and underlined for the bottom strand.

b IPD, interpulse duration.

c ORFs, open reading frames.

### Data availability.

The *H. salifodinae* BOL5-1 genome sequence has been deposited in GenBank under the accession numbers CP069188 through CP069193. The raw data are available under the BioSample accession number SAMN17385152.
